# Postnatal Craniofacial Skeletal Development of Female C57BL/6NCrl Mice

**DOI:** 10.3389/fphys.2017.00697

**Published:** 2017-09-14

**Authors:** Xiaoxi Wei, Neil Thomas, Nan E. Hatch, Min Hu, Fei Liu

**Affiliations:** ^1^Department of Orthodontics, Jilin University School and Hospital of Stomatology Changchun, China; ^2^Department of Biologic and Materials Sciences and Division of Prosthodontics, University of Michigan School of Dentistry Ann Arbor, MI, United States; ^3^Department of Orthodontics and Pediatric Dentistry, University of Michigan School of Dentistry Ann Arbor, MI, United States

**Keywords:** cranial base, synchondroses, postnatal, craniofacial, mouse, bone, μCT, C57BL/6NCrl

## Abstract

The craniofacial skeleton is a complex and unique structure. The perturbation of its development can lead to craniofacial dysmorphology and associated morbidities. Our ability to prevent or mitigate craniofacial skeletal anomalies is at least partly dependent on our understanding of the unique physiological development of the craniofacial skeleton. Mouse models are critical tools for the study of craniofacial developmental abnormalities. However, there is a lack of detailed normative data of mouse craniofacial skeletal development in the literature. In this report, we employed high-resolution micro-computed tomography (μCT) in combination with morphometric measurements to analyze the postnatal craniofacial skeletal development from day 7 (P7) through day 390 (P390) of female C57BL/6NCrl mice, a widely used mouse strain. Our data demonstrates a unique craniofacial skeletal development pattern in female C57BL/6NCrl mice, and differentiates the early vs. late craniofacial growth patterns. Additionally, our data documents the complex and differential changes in bone parameters (thickness, bone volume, bone volume/tissue volume, bone mineral density, and tissue mineral density) of various craniofacial bones with different embryonic origins and ossification mechanisms during postnatal growth, which underscores the complexity of craniofacial bone development and provides a reference standard for future quantitative analysis of craniofacial bones.

## Introduction

The craniofacial skeleton is distinct from the body skeleton in the complexity of its organization, its embryonic origin, and the molecular mechanisms inducing skeletogenesis. Craniofacial skeletal malformations represent one of the largest classes of birth defects (Warman et al., 2011). They compromise not only the esthetics and function, but also the mental wellbeing of the affected individual. However, most of our information about skeletal development comes from analyses of the postcranial skeleton. A thorough understanding of normal craniofacial skeletal development is required for a better understanding and treatment of defective craniofacial skeletal development.

The craniofacial skeleton is formed through two distinct ossification mechanisms (Kronenberg, 2003). Most of the craniofacial bones such as the calvaria, some facial bones, and mandible are formed through intramembranous ossification. On the other hand, the cranial base, a supporting platform for the development of the brain, is formed through endochondral ossification similar to that seen in the appendicular and axial skeletons. The cranial base contains multiple growth centers called synchondroses, which are the counterpart of growth plates in long bones. Synchondroses are comprised of mirror-image growth plates with a central resting zone surrounded by proliferative and hypertrophic zones at both sides (Wei et al., 2016). The spheno-occipital synchondrosis (SOS) joins the basi-sphenoid and occipital bones, and the intersphenoid synchondrosis (ISS) joins the pre-sphenoid and basi-sphenoid bones in cranial base. The ISS and SOS contribute significantly to the growth of both the cranium and the upper face (Wei et al., 2016). The premature fusion of either the ISS or the SOS can result in midface hypoplasia as seen in patients with syndromic craniosynostosis (Goldstein et al., 2014).

Craniofacial skeletons have two embryonic origins (Chai et al., 2000; Jiang et al., 2002). The majority of the craniofacial bones, including all facial bones, most cranial bones such as the frontal bones, temporal bones, and ethmoid bone, are derived from neural crest. In contrast, the parietal bone of the calvaria and the occipital bone are derived from mesoderm. It is known that embryonic origin has significant impact on osteoblast proliferation and differentiation. Neural crest-derived calvarial osteoblasts from the frontal bone have superior intrinsic osteogenic potential and tissue regeneration ability compared to mesoderm-derived calvarial osteoblasts from parietal bone (Quarto et al., 2010). Our previous studies suggested that there is increased bone volume and density in mesoderm derived parietal bone compared to neural crest derived frontal bone at early postnatal stage in both BALB/c congenic mice at 1 month of age (Liu et al., 2013) and 50% C57BL/6-50%129SF2/J mice at 2 weeks of age (Liu et al., 2015). However, a thorough knowledge of quantitative differences in bone parameters during postnatal development between neural crest-derived skull bone and mesoderm-derived skull bone is lacking.

The mouse model plays critical roles in unraveling the mechanisms of skeletal and craniofacial development and dysmorphology. Various mouse models have been available for craniofacial study for decades, and more models are being generated (Murray, 2011). Because key craniofacial developmental processes are similar across mice and humans (Martinez-Abadias et al., 2012), mouse models are great tools to study the pathological processes of craniofacial development. Importantly, the efficient and meaningful utilization of various mouse models depends on the thorough understanding of the normal craniofacial development of mice. However, detailed data on the postnatal craniofacial skeletal development of different mouse strains remains largely unavailable. C57BL/6NCrl mice are one of the most commonly used inbred mouse strains. A recent report elegantly documented the postnatal three-dimensional metrics of the skull in male C57BL/6J mice (Vora et al., 2016), which provided a reference standard for quantitative analysis up to nearly 4 months of age. It is known that gender has a significant impact on skeletal development (Gilsanz et al., 1997) and orofacial measurements (Nascimento et al., 2013). Thus, it is necessary to have the counterpart knowledge of female C57BL/6NCrl mice. Additionally, age related and bone specific changes in craniofacial bone parameters such as bone volume, bone volume/tissue volume, bone mineral density, and tissue mineral density in normal mouse development is lacking. Furthermore, while most mouse strains reach peak body bone mass around 4–6 months of age, there can be additional bone mass accrual between 6 and 12 months of age (Jilka, 2013). Importantly, human craniofacial skeleton has significant changes during adulthood (Israel, 1977; Pecora et al., 2008). Thus, it is necessary to characterize the dynamic changes in craniofacial skeletal development in adult mice. In this study, we employed high-resolution micro-computed tomography (μCT) to analyze in detail the craniofacial skeletal development of female C57BL/6NCrl mice up to 13 months of age. These analyses included three-dimensional skull metrics and individual bone parameters of neural crest-derived and mesoderm-derived, as well as intramembranous and endochondral craniofacial bones. The data demonstrates the unique and complex morphological and structural characteristics of the craniofacial skeleton of C57BL/6NCrl female mice during postnatal development. This information can serve as a reference standard for studies using this mouse strain to model abnormalities in craniofacial skeletal development as well as for the comparison with other mouse strains of different genetic background.

## Materials and methods

### Animals

C57BL/6NCrl (strain code 027; Charles River, MA) mice were used in this study. Mice were raised in an optimal and controlled environment with standard temperature and humidity at the University of Michigan School Of Dentistry. All animal handling protocols were approved by IACUC at the University of Michigan. The breeders were fed with pelleted rodent diets optimal for breeding units (LabDiet 5008). Pups were weaned at postnatal day P21 and fed with pelleted stock diet (LabDiet 5LOD). Mice were euthanized at designated postnatal ages (P0, P7, P14, P21, P30, P60, P90, P120, P180, P390, *n* = 4 in each age group) via CO_2_ exposure (older than P10) or decapitation (younger than P10). Age groups of P0, P7, P14, P21, P30, P60, P120 and number of mice per age group were selected based on a similar study using male C57BL/6J mice (Vora et al., 2016). P180 and P390 age groups were selected to include the adult mice after skeletal maturation.

### μCT scanning, reconstruction, orientation, and landmark placement

**Figure 1:**
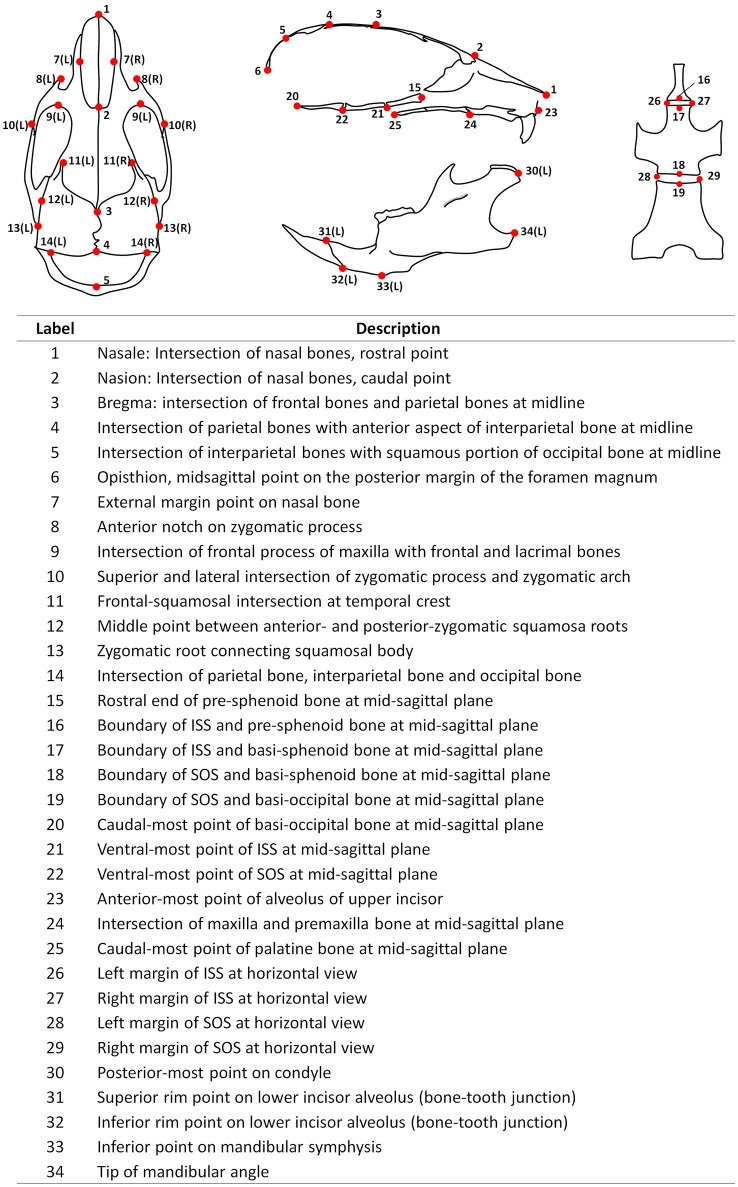
Diagrammatic mouse skull landmarks and descriptions.

Fixed mouse skulls were scanned in water using cone beam computed tomography (eXplore Locus SP, GE Healthcare Pre-Clinical Imaging, London, ON, Canada). Scan parameters included a 0.5° increment angle, 200 degree, 4 frames averaged, 1,600 ms exposure time, 2 × 2 detecting binning, an 80 kVp, 6.4 W power and 80 μA X-ray source with a 0.508 mm Al filter to reduce beam hardening artifacts, and a beam flattener around the specimen holder (Meganck et al., 2009). All images were reconstructed at an 18 μm isotropic voxel size and calibrated once daily to a calibration phantom of air, water and hydroxyapatite (1.69 mg/cc). Images were oriented and reconstructed to 3D via Microview (version ABA 2.2) and ITK-SNAP (version 3.6). To analyze the antero-posterior growth, a best-fit mid-sagittal plane was manually chosen and the coronal section was simultaneously adjusted to be most symmetrical. The rostral point of nasale and caudal point of basi-occipital bone were set on the same horizontal plane at the mid-sagittal section. Landmarks (21 single, 13 paired, total 47, see Figure 1) and linear measurements (44 items, see Table 1) were used to analyze mouse skull morphology. Most of these parameters have been defined previously (Richtsmeier et al., 2000; Vora et al., 2016). At P7 and P14, some landmarks (labeled 3, 4, and 5) were difficult to identify due to unclosed anterior and posterior fontanelles. Thus, we did not include the measurements related to these landmarks at early postnatal stages (② ③ ④ at P7, and ③ ④ at P14). All the parameters were measured twice by the same investigator and the average value was used.

**Table 1:** List of linear and angle measurements used in this study.

**Type**	**Label**	**Landmarks**	**Description**
Anterior-posterior	①	1-2	Length of nasal bone
	②	2-3	Length of frontal bone
	③	3-4	Length of parietal bone
	④	4-5	Length of interparietal bone
	⑤	5-6	Length of occipital bone
	⑥	1-6	Skull length
	⑦	2-6	Cranial length
	⑧	1-25	Facial length
	⑨	2-15	Anterior cranial base length
	⑩	15-20	Posterior cranial base length
	⑪	15-16	Length of pre-sphenoid bone
	⑫	17-18	Length of basi-sphenoid bone
	⑬	19-20	Length of basi-occipital bone
	⑭	16-17	Length of ISS
	⑮	18-19	Length of SOS
	⑯	12-23	Length of upper jaw
Transversal	⑰	7(L)-7(R)	Nasal width
	⑱	8(L)-8(R)	Anterior zygomatic width (facial width)
	⑲	9(L)-9(R)	Inter-orbital width
	⑳	10(L)-10(R)	Inter-zygomatic arches width
	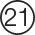	11(L)-11(R)	Frontal width
	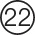	13(L)-13(R)	Anterior zygomatic width (skull width)
	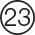	14(L)-14(R)	Interparietal width
	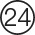	26-27	Width of ISS
	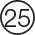	28-29	Width of SOS
Vertical	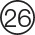	2-24	Facial height
	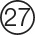	3-21	Anterior-cranial height
	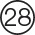	4-22	Mid-cranial height (skull height)
	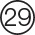	5-20	Posterior-cranial height
Mandible	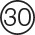	30-31	Upper mandibular length
	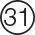	33-34	Lower mandibular length
	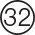	30-34	Mandibular height
	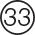	34(L)-34(R)	Mandibular width
Angular	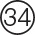	21-2-3	Rostral angle of cranium cavity
	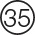	2-3-4	Anterior-middle cranial vault angle
	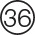	3-4-5	Mid-posterior cranial vault angle
	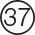	4-5-6	Posterior cranial vault angle
	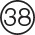	5-6-20	Caudal angle of cranium cavity
	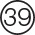	6-20-21	Cranial vault-cranial base angle
	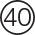	20-21-2	Cranial base angle
	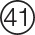	25-2-1	Snout angle
	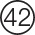	21-2-23	Cranium-maxilla angle
	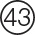	21-2-31	Cranium-mandible angle
	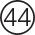	23-2-31	Maxilla-mandible angle

### Linear and angular measurement

Following euthanasia and weight measurement, the body length, skull length, and tail length with skin and soft tissue were measured by digital caliper (Fowler 6″/150 mm Ultra-Cal V, Sylvac SA) (Figure S1A). The body length was defined as the distance between the tip of the nose and the base of the tail, the skull length was defined as the distance between the tip of the nose and the junction of skull and cervical vertebrae, and the tail length was defined as the distance between base of the tail and tip of the tail. Femur length and tibia length were obtained after dissection. In addition, 33 linear measurements (16 anterior-posterior, 9 transversal, and 4 vertical, as well as 4 mandible parameters) and 11 angular measurements were measured using skull μCT files. We used Euclidian distances to do the measurements except using the projected distance for a few anterior-posterior (A-P) dimensions (⑥ ⑦ ⑧ ⑨ ⑩). Bilateral symmetrical parameters (⑯ 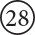 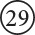 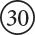) were recorded by averaging the results of both sides. Angular measurements and the cranial cavity area were obtained at the mid-sagittal plane using ImageJ (version 1.4.3.67). To obtain the relative position and growth pattern of jaws to the cranium as well as the relative position of the maxilla to the mandible, we collected the correlative angles that are equivalent to those in human measurements.

### μCT analysis of bone parameters

All μCT analysis was performed using the Microview (version ABA 2.2) analysis software. Similar to our previous work (Liu et al., 2013, 2015), a region of interest (ROI) was selected for each of the bones to be analyzed (frontal, parietal, pre-sphenoid, basi-sphenoid, basi-occipital and mandible) (Figures S1B–D). For the frontal bone, a 0.5 mm × 0.5 mm area was located 1.5 mm anterior to the intersection point of coronal suture and sagittal suture and 1 mm lateral to the posterior frontal suture. For the parietal bone, a 0.5 mm × 0.5 mm area was located 1.5 mm posterior to the intersection point of coronal suture and sagittal suture and 1.5 mm lateral to the sagittal suture. The entire thickness of both the frontal and parietal bones was selected for measurement. For the pre-sphenoid bone, we first identified a plane which is 0.09 mm anterior to the anterior border of ISS and perpendicular to the long axis of the bone, and then selected the entire bone structure 0.5 mm anterior to this plane. For the basi-sphenoid bone, we first identified a sagittal plane which is parallel to the long axis and in the center of this bone; and starting from the middle line of basi-sphenoid bone at sagittal plane, an area with 0.5 mm in length and full thickness of bone in height was defined; similar areas are defined in all the sagittal sections 0.25 mm adjacent to the initial sagittal plane (thus ROI includes a center 0.5 mm in width in coronal sections). For the basi-occipital bone, we first identified a coronal plane which is 0.09 mm posterior to the posterior border of the SOS and perpendicular to the long axis of the bone; and then an area of 0.5 mm in length and full thickness of bone in height was chosen lateral to the mid-sagittal plane. Similar areas are defined in all the sections 0.5 mm posterior to the initial coronal plane. For the mandible, we first identified the inferior point of the lingula mandibulae in the coronal section, and then an area of 0.5 mm (from the inferior point of the lingula mandibulae toward the inferior border of mandible) times full thickness of bone was chosen. Similar areas are defined in all the sections 0.5 mm posterior to the initial plane. The ROI defined by this method was able to avoid the teeth and generate repeatable measurements. After the ROI was selected, bone parameters (volume, volume of bone [BV], bone mineral density [BMD], tissue mineral density [TMD], bone volume/total volume [BV/TV] and thickness) were calculated after applying a fixed threshold. For each bone at different developmental stage, a uniform threshold was applied to calculate bone parameters (Table 2).

**Table 2:** Threshold for each bone at different developmental stages (Hounsfield Units).

	**P14**	**P21**	**P30**	**P60**	**P90**	**P120**	**P180**	**P390**
Frontal	1,000	1,600	1,800	1,800	1,800	1,800	1,800	1,600
Parietal	1,000	1,800	1,800	2,000	1,800	1,800	1,800	1,600
Pre-sphenoid	800	1,000	1,000	1,200	1,400	1,400	1,400	1,200
Basi-sphenoid	800	1,000	1,000	1,200	1,200	1,400	1,400	1,400
Basi-occipital	600	1,000	1,000	1,400	1,400	1,400	1,400	1,400
Mandible	1,000	2,000	2,000	2,400	2,400	2,400	2,400	2,400

### Statistical analysis

One way ANOVA with Tukey's multiple comparison tests were performed for the comparisons among different time points in each parameter using GraphPad Prism 7.0 (GraphPad, San Diego, CA, USA). ^*^p < 0.05 was considered to be significant and data were presented as the mean ± SD.

## Results

### Postnatal growth in the C57BL/6NCrl mouse

**Figure 2:**
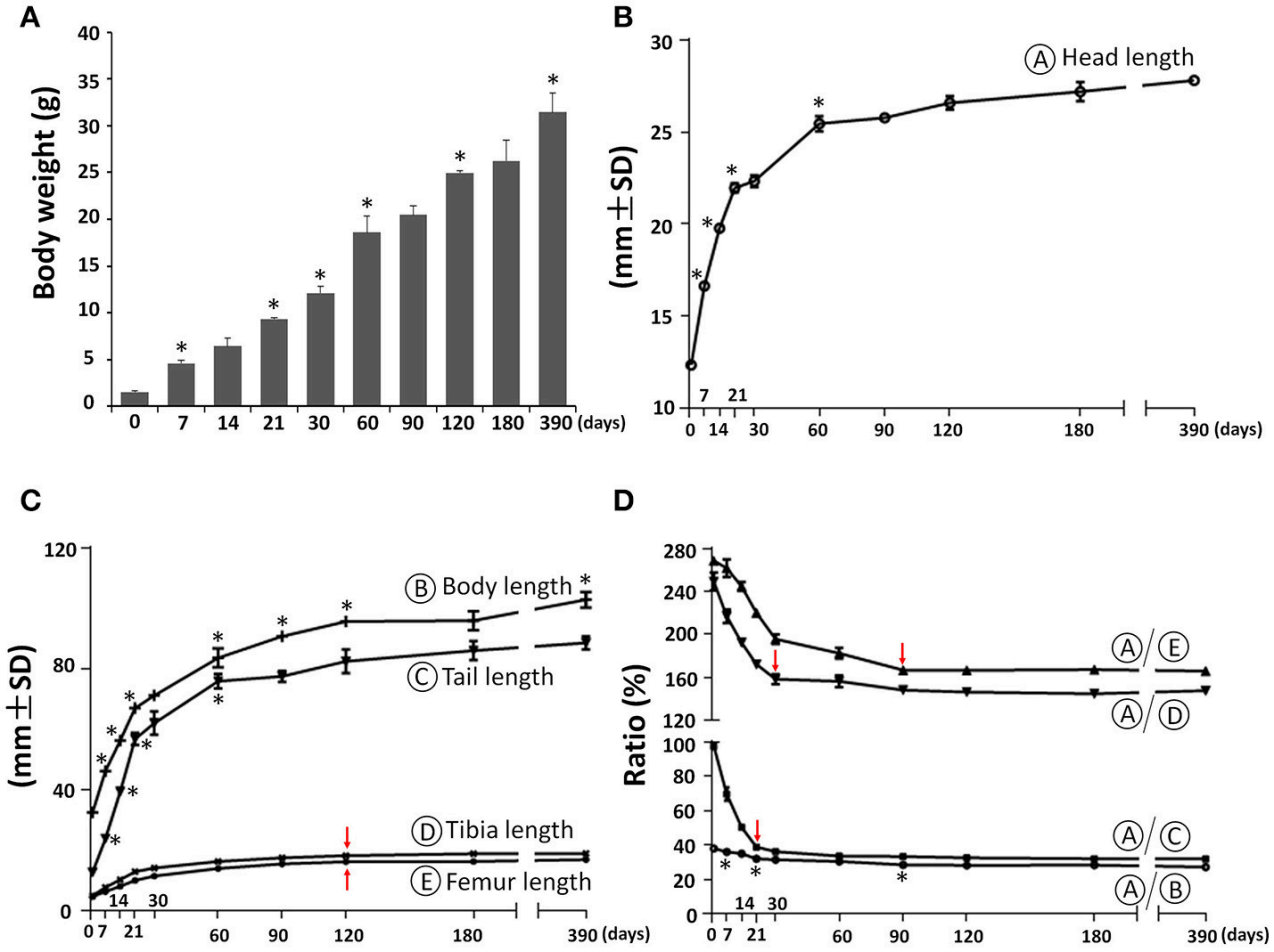
Postnatal skeletal growth in female C57BL/6NCrl mice. **(A)** Body weight. **(B)** Head length (circled A). **(C)** Body length, tail length, tibia length, and femur length (circled B, C, D, E, respectively). **(D)** Ratio between the length of head and other parts of the body. ^*^Represents significant change between indicated data point and immediate earlier time point, and red arrows indicate significant changes between all the neighboring time points before that time point (Tukey's multiple comparison test, *p* < 0.05).

The body weight of C57BL/6NCrl female mice increased postnatally with the highest daily increase during the first week at 0.44 g/day, followed by increases between P7 and P60 at 0.26 g/day, increases between P60 and P120 at 0.11 g/day and the lowest daily increase after P120 at 0.02 g/day (Figure 2A). As a first step to determine the postnatal craniofacial skeletal growth of C57BL/6NCrl female mice, we measured the head length including soft tissue using a digital caliper (Figure 2B). The head length had the fastest growth during the first week at 0.62 mm/day, with additional fast growth between P7 and P21at 0.38 mm/day, followed by moderate growth between P21 and P60 at 0.09 mm/day, significantly decreased growth between P60 and P180 at 0.016 mm/day, and nearly no growth between P180 and P390 at 0.003 mm/day. In order to determine the relationship between head growth and other parts of the body skeleton, we measured the length of whole body, tail, tibia, and femur (Figure 2C) and calculated the relative ratio between head length and these parts at P0, P7, P14, P21, P30, P60, P90, P180, and P390 (Figure 2D). Similar to the head, the length of whole body, tail, tibia, and femur had the greatest growth during the first 3 weeks after birth, whereas growth after 2 months of age was very slow (Figure 2C). Interestingly, the ratio between the length of the head and other parts of the skeleton was greatest at birth. This ratio then greatly decreased until P30, and plateaued after P90 (Figure 2D), indicating relatively greater growth of the skull before birth, but faster growth of other parts of the body during the first postnatal month, and then proportionate growth of whole body and head afterwards, which is similar to human growth (Huelke, 1998; Proffit et al., 2007).

### Postnatal craniofacial skeletal growth in the C57BL/6NCrl mouse

**Figure 3:**
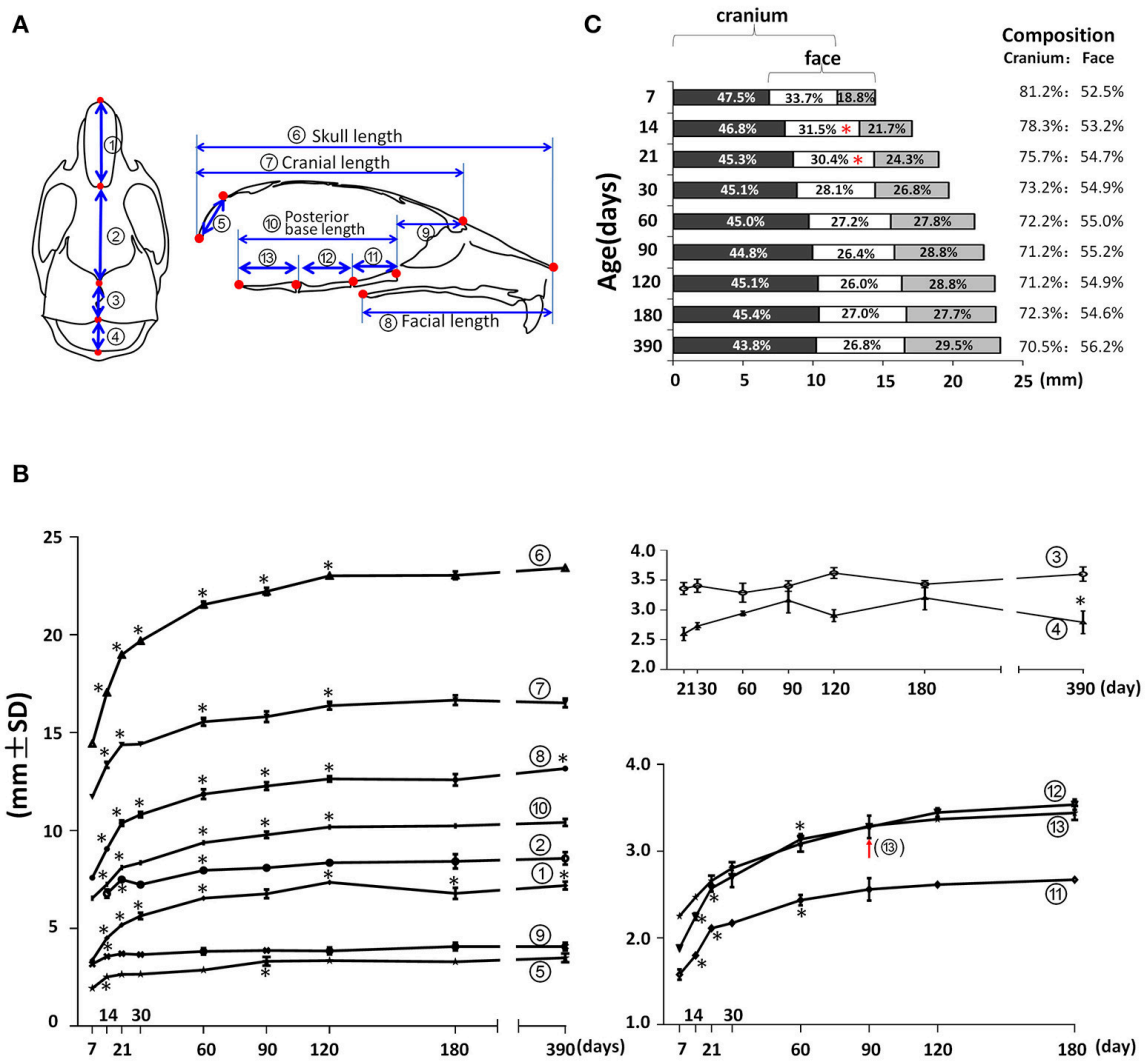
Anterior-posterior (A-P) growth of the mouse craniofacial skeleton. **(A)** Diagram of mouse skull at ventral view (left) and mid-sagittal plane (right) with landmarks and parameters used for A-P dimension measurements. **(B)** Growth curves showing dynamic change of individual parameters on A-P dimension with age (landmarks and labels were described in Figure 1 and Table 1). **(C)** Bar graph showing the growth of the cranial region, facial region, and the overlap region during skull development. Numbers in the bar graph indicate the proportion of each region: cranium exclusive of overlap, overlap, face exclusive of overlap; numbers on the right indicate the proportions of the cranium and face including the overlap. ^*^Represents significant change between indicated data point and immediate earlier time point, and red arrow indicates significant changes between all the neighboring time points before that time point (Tukey's multiple comparison test, *p* < 0.05).

In the mid-sagittal plane of reconstructed mouse skull μCT images, the overall A-P length as well as the contained cranial and facial regions demonstrated a continuous increase until P120 (Figures 3A,B; circled 6, 7, and 8 respectively). The nasal bone, frontal bone, parietal bone, interparietal bone, and occipital bones contributed to the overall A-P length of the skull. The length of nasal bone (Figures 3A,B; circled 1) had the fastest postnatal growth before 1 month of age and continued to increase until P120. Interestingly there was an 8% decrease between P120 and P180 and a 6% increase between P180 and P390 in the nasal bone length, suggesting continuous remodeling of the nasal bone at a later developmental stage. The length of the frontal bone (Figures 3A,B; circled 2) had the fastest increase before 3 weeks of age; there was continuous incremental increase until P390 with a 6% increase from P90 to P390 (*p* < 0.05). The length of the parietal bone (Figures 3A,B; circled 3) remained relatively constant at early postnatal stage until a 10% increase between P60 and P120 (*p* < 0.05). The growth curve of the interparietal bone (Figures 3A,B; circled 4) was complementary to that of the parietal bone; it had a 16% increase between P30 and P90 and a 13% decrease between P180 and P390 (*p* < 0.05). The length of the occipital bone (Figures 3A,B; circled 5) had the fastest increase before 2 weeks of age and continued to increase until P90. The middle-sagittal overall A-P length can be divided into cranial and facial regions (Figure 3A; circled 7 and 8). Noticeably, there was a small but statistically significant 4% increase in facial length (Figure 3B; circled 8) between p180 and P390, which was likely due to growth in the nasal-maxilla complex during this period. The length of the anterior cranial base (Figures 3A,B; circled 9) had an increase only between P7 and P14. The length of the posterior cranial base (Figures 3A,B; circled 10) continued to increase until P120 with similar contributions from the three individual bones of the posterior cranial base: the pre-sphenoid, basi-sphenoid and basi-occipital bones (Figures 3A,B; circled 11, 12, and 13 respectively). The antero-posterior dimension of the skull is increased through growth of facial, cranial and overlapping regions (Figures 3A,C), and the composition of this dimension has a dynamic change during age progression. At P7, the cranial region (including the overlap) constituted 81.2% of the total A-P dimension, gradually decreased to 72.2% at P60 and remained until P180, and further decreased to 70.5% at P390. In contrast, the facial region (including the overlapping region) constituted 52.5% of the total A-P dimension at P7, increased to 55.0% at P60 and remained until P180, and there was a further slight increase to 56.2% at P390. The overlap region contributed to 33.7% of the A-P dimension at P7, gradually decreasing to 26.4% at P90. Thus, during postnatal development, the facial region had a greater increase in A-P length compared to the cranial region, and this different growth pattern even occurred at very late development stages, such as between P180 and P390.

**Figure 4:**
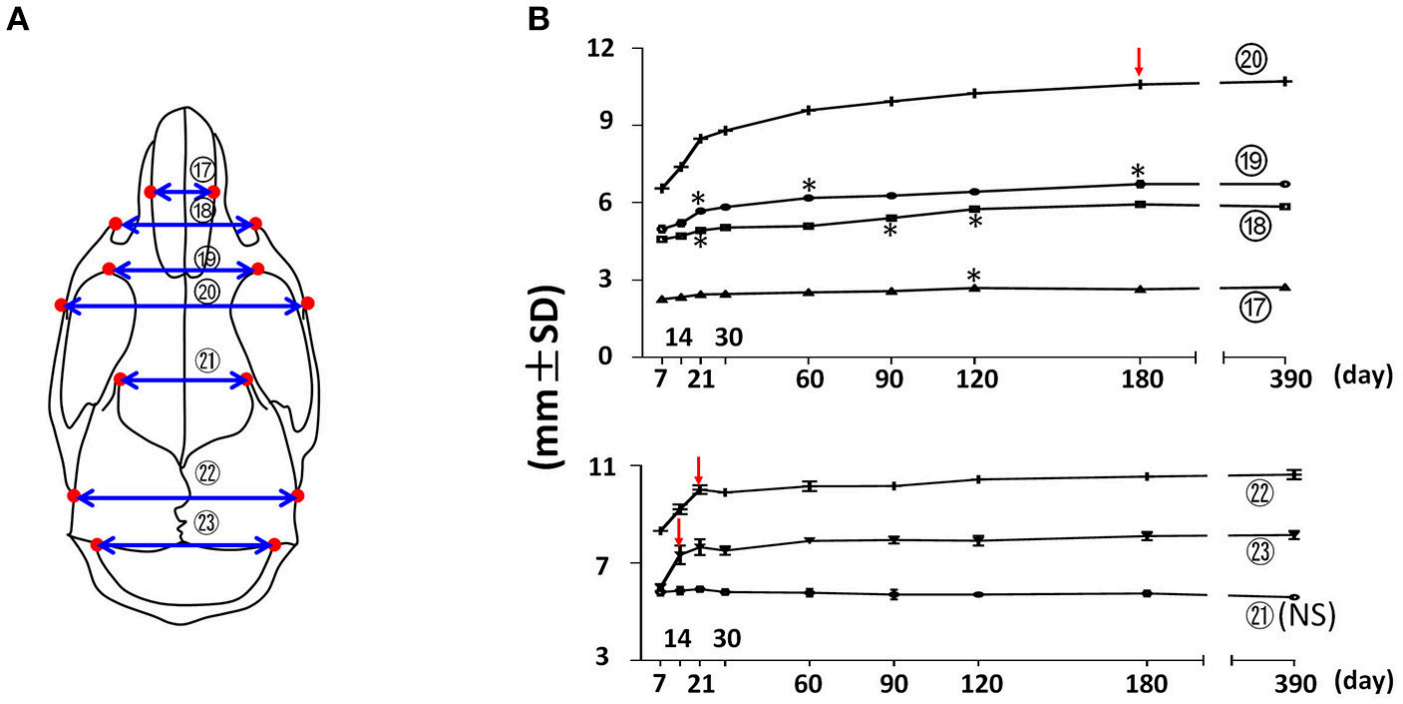
Transverse growth of the mouse craniofacial skeleton. **(A)** Diagram of mouse skull at ventral view with landmarks and parameters used for transverse dimension measurements. **(B)** Growth curves showing changes in transverse dimension with age (landmarks and labels were described in Figure 1 and Table 1), including 4 parameters in facial region (upper) and 3 parameters in cranial region (lower). ^*^Represents significant change between indicated data point and immediate earlier time point, and red arrows indicate significant changes between all the neighboring time points before that time point (Tukey's multiple comparison test, *p* < 0.05). NS indicates that there was no statistical significance among the data points.

The width of the facial region was quantified by nasal width, facial width (anterior zygomatic width), inter-orbital width, and inter-zygomatic arch width (Figure 4; circled 17, 18, 19, and 20 respectively). The nasal width (Figure 4; circled 17) demonstrated a continuous, very small incremental increase with age after P7 until P120. The facial width (Figure 4; circled 18) showed a similar pattern, but with a slightly larger magnitude and a peak at P180. More posteriorly, the inter-orbital width (Figure 4; circled 19) and the inter-zygomatic arch width (Figure 4; circled 20) showed a similar trend of continuously increasing growth with age, but at a larger magnitude. Together, these data suggest that the width of the facial region continues to increase with age steadily, and that there was bigger increase in the posterior facial region than in the anterior region after P7. This indicates that the width of the anterior facial region is established mainly before P7. The width of the cranial region was quantified by the frontal width, inter-posterior zygomatic width, and interparietal width (Figure 4; circled 21, 22, and 23 respectively). The frontal width had no significant difference at the examined time points, suggesting that the width of anterior cranial region is established before P7. In contrast, the posterior zygomatic width and interparietal width had the most significant increase during the first 3 weeks after birth, with very minor changes afterwards. Altogether, these data suggest that transverse dimensions of the posterior facial and cranial regions have a continuous increase after birth with age. In contrast, the transverse dimensions of the anterior facial and cranial regions are established before 1 week of age in the female C57BL/6NCrl mice.

**Figure 5:**
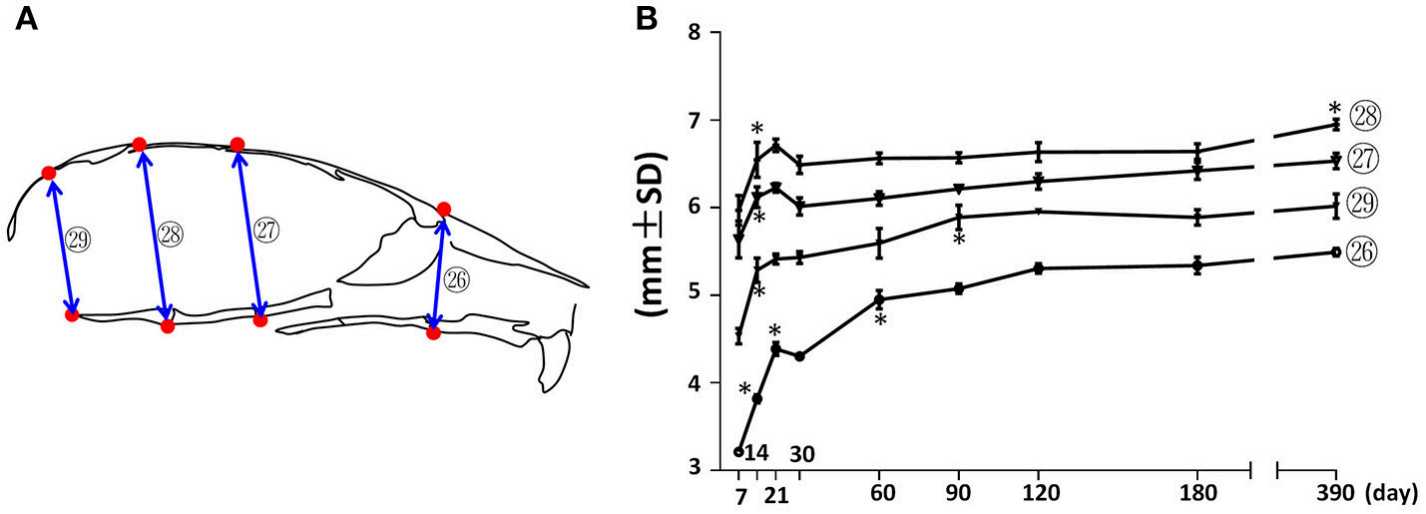
Vertical growth of the mouse craniofacial skeleton. **(A)** Diagram of mouse skull at mid-sagittal plane with landmarks and parameters used for vertical dimension measurements. **(B)** Growth curves showing dynamic change of individual parameters on vertical dimension with age (landmarks and labels were described in Figure 1 and Table 1). ^*^Represents significant change between indicated data point and immediate earlier time point (Tukey's multiple comparison test, *p* < 0.05).

Vertical measurements were made using the mid-sagittal landmarks in the facial and cranial regions (Figure 5). The facial height (Figure 5; circled 26) had the fastest increase (37%) between P7 and P21, followed by a 23% increase between P30 and P120. The height of the vault was quantified by the anterior-cranial height, middle-cranial height, and posterior-cranial height (Figure 5, circled 27, 28, and 29 respectively). The height of the cranial vault increased significantly between P7 and P14, following by changes that were much smaller in magnitude. Interestingly, although there was no other significant increase in anterior-cranial height and posterior-cranial height after P90, there was still a small but statistically significant 5% increase in the middle-cranial region between P180 and P390.

**Figure 6:**
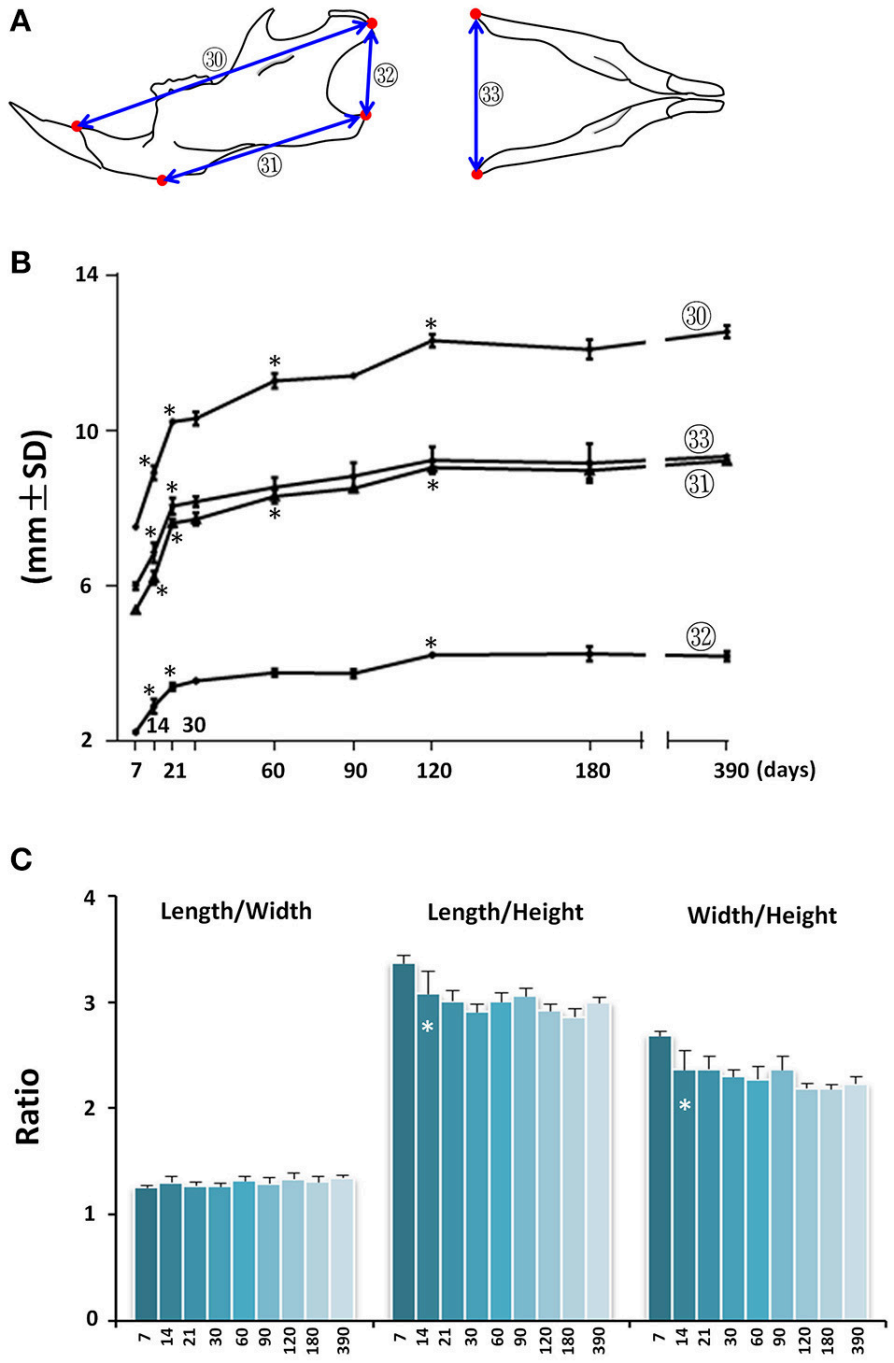
Postnatal growth of the mandible in three dimensions. **(A)** Diagrammatic lateral and inferior view of mouse mandible with landmarks and parameters used for mandible measurements. **(B)** Growth curves showing dynamic change of individual mandible parameters with age (landmarks and labels were described in Figure 1 and Table 1). **(C)** Ratio of mandibular growth in three dimensions. ^*^Represents significant change between indicated data point and immediate earlier time point (Tukey's multiple comparison test, *p* < 0.05).

Mandibular growth was evaluated by upper and lower anterior-posterior length (Figure 6A; circled 30 and 31 respectively), ramus height (Figure 6A, circled 32), and inter-angular width of mandible (Figure 6A; circled 33). The anterior-posterior growth of the mandible was greatest between P7 and P21, followed by a smaller increase through P120 (Figure 6B; circled 30 and 31). The vertical dimension of the mandible measured at the posterior ramus region also had the greatest increase between P7 and P21, followed by incremental increases until P120 (Figure 6B; circled 32). The inter-angular width of mandible (Figure 6B; circled 33) had a similar growth pattern as that of the anterior-posterior length, with the greatest increase between P7 and P21. Of note, there was a 52% increase in mandibular height between P7 and P21. However, there was only a 17% increase in lower mandibular length, 36% increase in upper mandibular length, and 34% increase in mandibular width, indicating a greater rate of vertical growth during this period. Consistent with this differential three-dimensional growth pattern in the mandible, the length/height ratio and width/height ratio decreased after P7, but the length/width ratio remained the same (Figure 6C).

### Proportional changes of the craniofacial skeleton during postnatal development

The morphology of the craniofacial complex is the result of coordinated three-dimensional growth, and the proportional growth of different dimensions is the key determinant of skull shape. To further characterize craniofacial skeletal growth of female C57BL/6NCrl mice, we determined the proportional growth change among different portions of the craniofacial bones.

**Figure 7:**
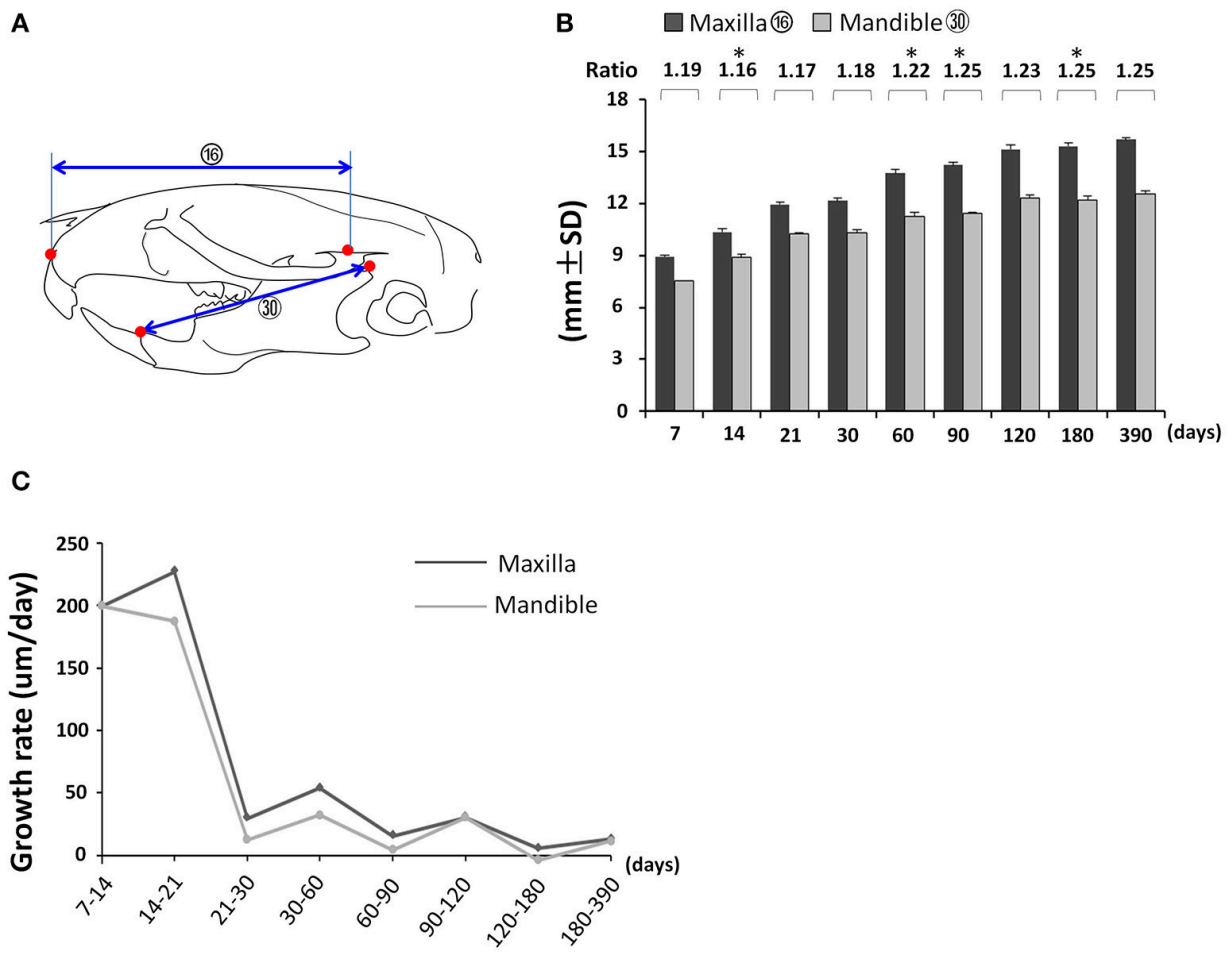
Coordinated growth of the maxilla and mandible during postnatal development. **(A)** Diagram of lateral view of mouse skull with landmarks and parameters used for the measurement of maxilla and mandible. **(B)** Changes in length of maxilla and mandible with age and the ratio between them. **(C)** Growth rate of maxilla and mandible between each designated age intervals. ^*^Represents significant change between indicated data point and immediate earlier time point (Tukey's multiple comparison test, *p* < 0.05).

First, we analyzed the proportional growth of the maxilla and mandible by comparing the relative length of maxilla (Figure 7A; circled 16) and mandible (Figure 7A; circled 30). The mandible grew continuously until P120 but the maxilla kept growing afterwards (Figure 7B). The maxilla/mandible ratio was relatively constant from P7 to P30 except for a very small but statistically significant decrease from P7 to P14, followed by a slight increase after P60 (Figure 7B). Both the maxilla and mandible showed the greatest growth velocity during the first 21 days, and a sharp drop afterwards, reaching nearly zero by P120 (Figure 7C). The maxilla had a greater growth velocity than the mandible at all intervals before P90. The accumulated effect of this differential growth velocity likely explains the increase in maxilla/mandible ratio and more anterior position of the maxilla and mandible relative to the cranium at later time points.

**Figure 8:**
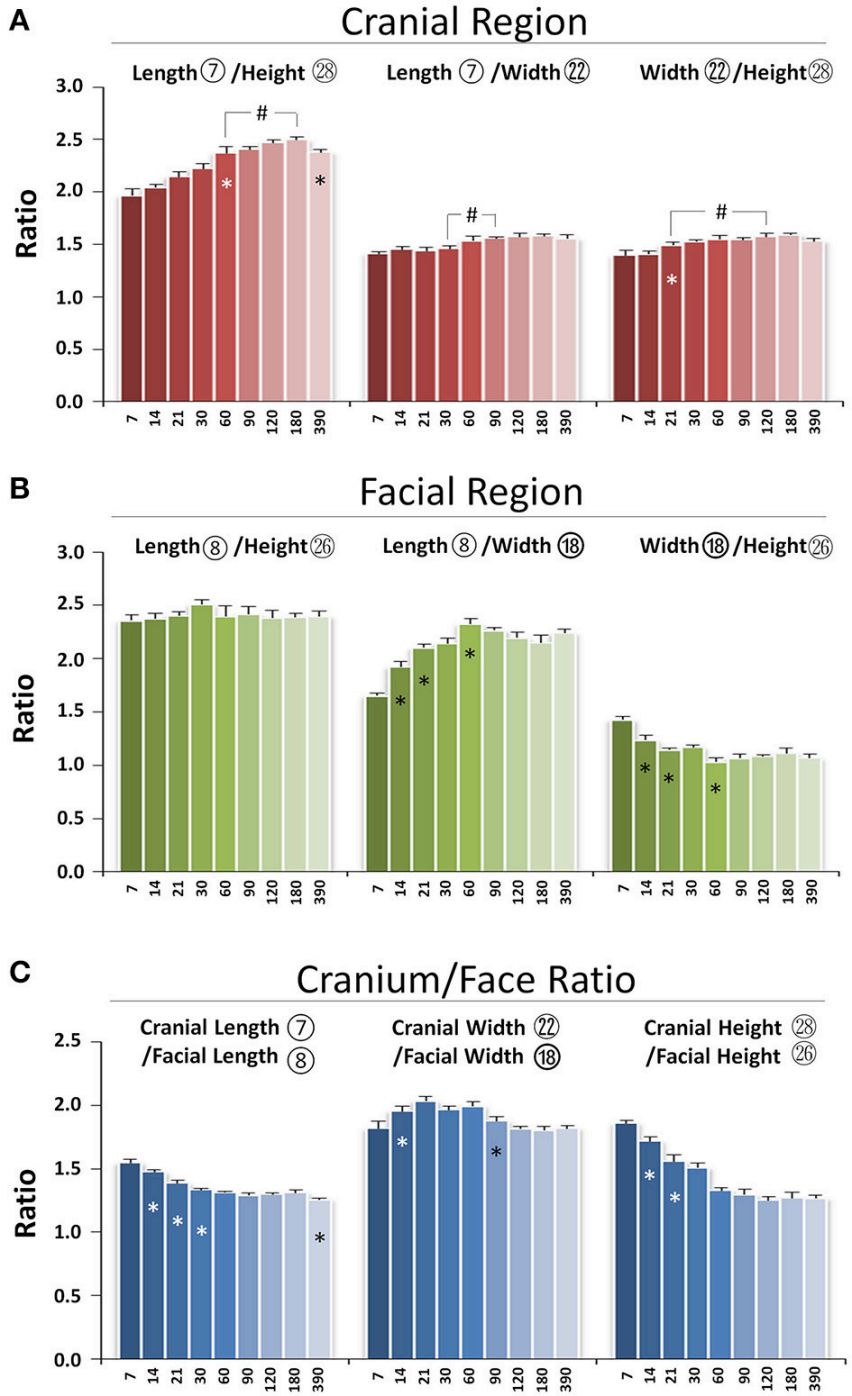
Proportional changes of craniofacial skeletal growth during postnatal development. **(A)** Ratio of three dimensions in cranial region. **(B)** Ratio of three dimensions in facial region. **(C)** Ratio between cranium and face in length, width, and height. ^*^Represents significant change between indicated data point and immediate earlier time point; ^#^Represents significant change between indicated data points (Tukey's multiple comparison test, *p* < 0.05).

The skull grows three-dimensionally in length, height, and width. In the cranial region, the length/height ratio was lowest at P7 and continued to increase until p180, indicating faster growth in cranial length compared to height postnatally. The length/width ratio remained the same from P7 to P30 with bigger values at P60 and afterwards, indicating proportional growth in cranial length and width during the first month after birth, followed by greater increases in length afterwards. The width/height ratio had an incremental increase from P21 to P60, reflecting faster growth in width compared to height during that period of development (Figure 8A). In the facial region, the length/height ratio had no significant change over time, indicating balanced postnatal growth in facial length and height. The facial length/width ratio had a gradual increase from P7 to P60, indicating faster growth in length compared to width. In contrast, the width/height ratio had a gradual decrease from P7 to P60, indicating slower growth in width compared to height (Figure 8B). Thus, in the cranial region, the order of early postnatal dimensional increases were length > width > height, while in the facial region, the order was length = height > width.

The skull is composed of cranial and facial regions with coordinated growth. The cranial/facial ratio in length, width, and height was always >1 (Figure 8C). The cranial/facial ratio in length continued to decrease from P7 to P30 and plateaued afterwards, indicating faster facial than cranial length growth at early postnatal stage. The cranial/facial ratio in width had an increase from P7 to P21 and a decrease from P60 to P120, indicating that width growth in facial region was slower in the first 3 weeks after birth but increased after P60. The cranial/facial ratio in height continued to decrease from P7 to P60, indicating relatively faster postnatal facial height growth during that developmental stage. Hence, the facial region had faster growth in both length and height compared to the cranial region during the first 2 months of age, but the width growth in the facial region was slower at early stage but faster at later stage.

### Angular changes of the craniofacial skeleton during postnatal development

**Figure 9:**
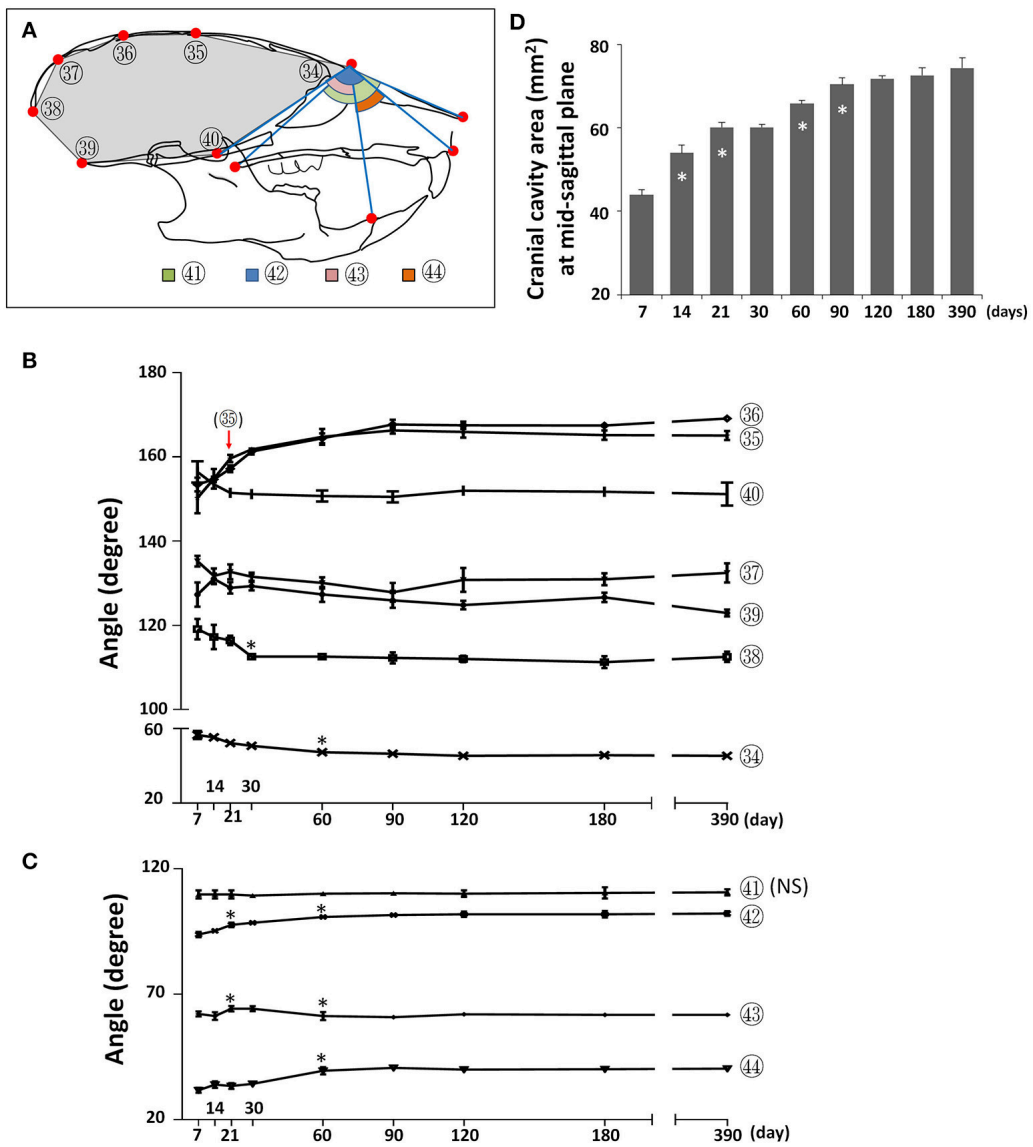
Angular changes during postnatal craniofacial growth. **(A)** Diagram of mouse skull at lateral view showing labels and parameters used for angular measurement and cranial cavity area measurement (gray area). **(B)** Angular changes of parameters in cranial region. **(C)** Angular changes of parameters in facial region. **(D)** Cranial cavity area measured at mid-sagittal plane with age. ^*^Represents significant change between indicated data point and immediate earlier time point, and red arrow indicates significant changes between all the neighboring time points before that time point (Tukey's multiple comparison test, *p* < 0.05). NS indicates that there was no statistical significance among the data points.

To assess changes to the skull shape during development, we evaluated the changes in angles between landmarks at the mid-sagittal plane. The cranial vault became flatter postnatally, as evidenced by a continuous increase in the mid-cranial angles (Figure 9; circled 35, 36) from P7 until P90 as well as an increase in the posterior-cranial angle (Figure 9; circled 37) from P7 to P21. In contrast, the cranial base became deeper, as evidenced by a decrease in two cranial base angles (Figure 9; circled 39, 40) from P7 to P21. The angle in the most anterior (Figure 9; circled 34) and most posterior (Figure 9; circled 38) regions became smaller from P7 to P30, indicating that the cranial cavity became more fusiform. A cumulative effect of skull shape change, the cranial cavity area had a continuous increase from P7 to P90, with the fastest increase between P7 and P14 (Figure 9D). The angle between the cranial base and maxilla (Figure 9; circled 42) as well as the angle between the mandible and maxilla (Figure 9; circled 44) gradually increased from P7 until P60, indicating more forward growth of maxilla relative to the cranial base and mandible. The facial angle (Figure 9; circled 41) showed no significant change during postnatal development and the cranium-mandible angle (Figure 9; circled 43) had some small but statistically significant changes during the first 2 months of age. Together, the data suggested that A-P growth of maxilla is the most prominent of the facial region postnatally in female C57BL/6NCrl mice.

### Postnatal craniofacial bone development

**Figure 10:**
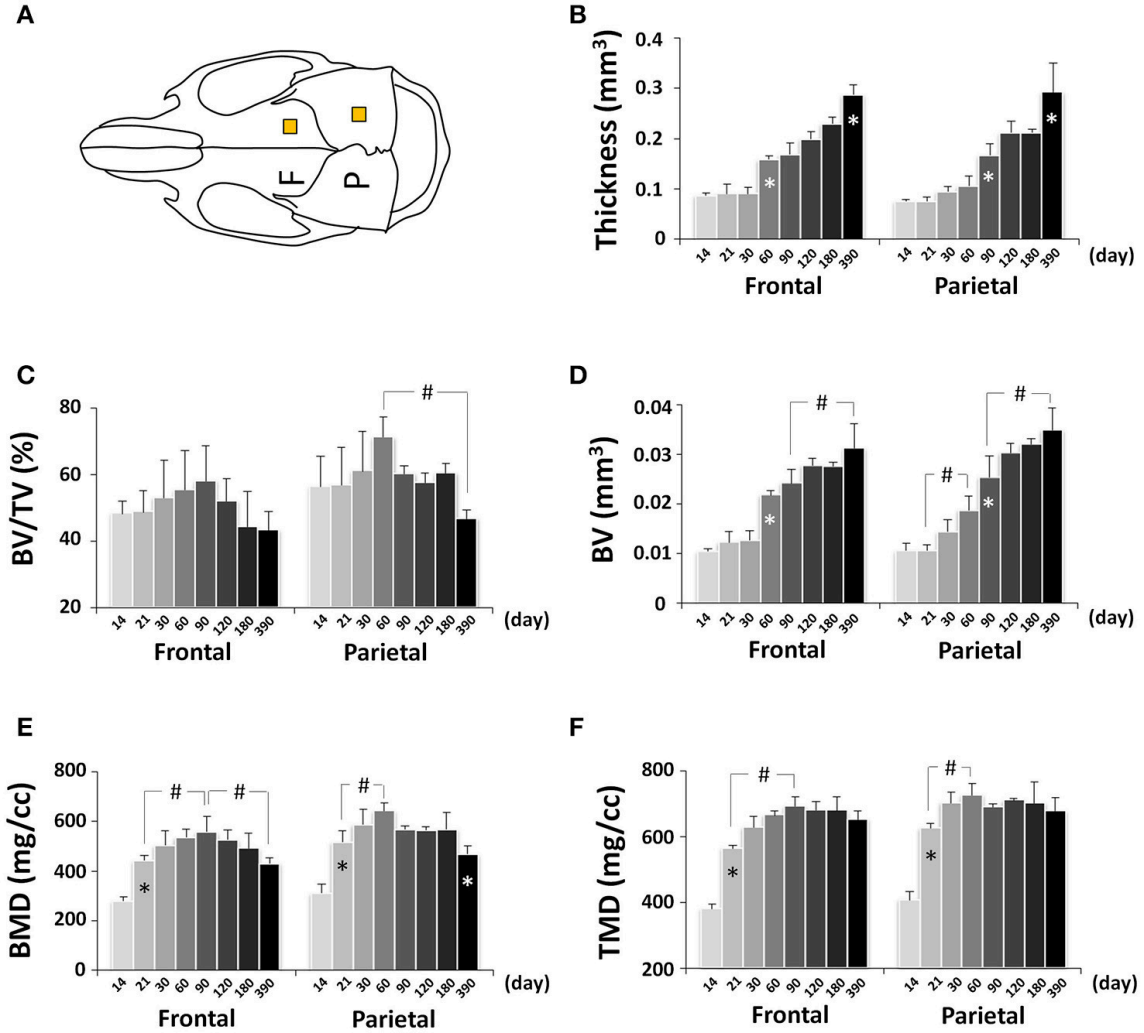
μCT analysis of bone parameters of cranial vault bones (frontal and parietal bone). **(A)** Diagram of mouse skull at dorsal view showing the measured area in frontal bone (F) and parietal bone (P). **(B–F)** μCT analysis of bone parameters (thickness, bone volume/tissue volume[BV/TV], bone volume[BV], bone mineral density[BMD], and tissue mineral density[TMD], respectively) in frontal and parietal bone with age. ^*^Represents significant change between indicated data point and immediate earlier time point; ^#^Represents significant change between indicated data points (Tukey's multiple comparison test, *p* < 0.05).

Craniofacial bone development can be altered in pathological conditions and mouse models are frequently used to determine the underlying mechanisms (Damazo et al., 2007; Liu et al., 2014; Fang et al., 2015a,b); however, there is lacking detailed knowledge of normative mouse craniofacial bone development. To determine the dynamic bone growth patterns in the cranial vault during postnatal development of C57BL/6NCrl mice, bone parameters of frontal and parietal bones were analyzed by μCT (Figure 10A; Figure S1A). In neural crest-derived frontal bone, the thickness remained at a similar value from P14 to P30 but continuously increased afterwards, with the quickest increase between P30 and P60 (Figure 10B); bone volume/tissue volume (BV/TV) peaked at P90 and subsequently decreased continuously (Figure 10C); bone volume (BV) had similar change pattern as thickness (Figure 10D); bone mineral density (BMD) peaked at P90 and decreased continuously afterwards (Figure 10E); tissue mineral density (TMD) peaked at P90 and decreased at P390 (Figure 10F). In mesoderm-derived parietal bone, the bone development dynamic was different from frontal bone. The quickest thickness increase occurred between P60 and P90 and there was no change between P120 and P180 (Figure 10B); BV/TV in parietal bone was significantly higher than that of frontal bone although the difference between two bones at each individual data point was not statistically significant; it peaked at P60 and significantly decreased at P390 (Figure 10C); the change in BV mirrored the change in thickness (Figure 10D); BMD peaked at P60 and significantly decreased at P390 (Figure 10E); TMD had the biggest increase from P14 to P21 and peaked at P60 (Figure 10F).

**Figure 11:**
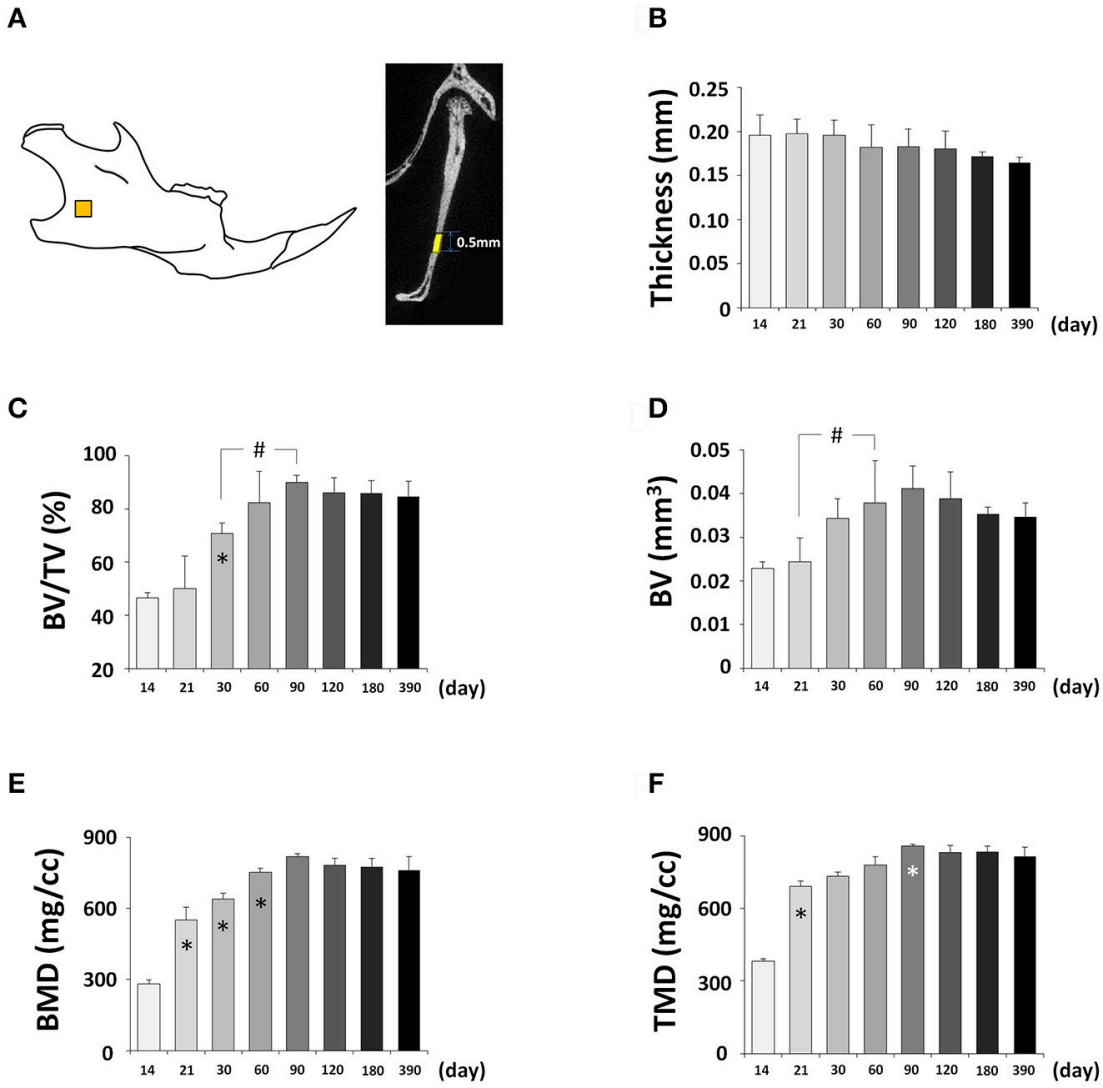
μCT analysis of bone parameters of mandible. **(A)** Diagram of mandible at lateral view showing the measured area. **(B–F)** μCT analysis of bone parameters (thickness, bone volume/tissue volume[BV/TV], bone volume[BV], bone mineral density[BMD], and tissue mineral density[TMD], respectively) in mandible with age. ^*^Represents significant change between indicated data point and immediate earlier time point; ^#^Represents significant change between indicated data points (Tukey's multiple comparison test, *p* < 0.05).

Next, we determined changes in bone parameters in the mandible of the C57BL/6NCrl mice during postnatal development. A defined area was employed to perform the quantitative mandibular micro CT measurements (Figure 11A, Figure S1D). The thickness of the mandible was decreasing over time but the changes were not statistically significant (Figure 11B). The BV/TV had a significant increase until P90 and then stabilized (Figure 11C). As a result, BV also had a significant increase until P90 (Figure 11D). BMD (Figure 11E), and TMD (Figure 11F) had the similar change pattern as BV. Interestingly, the time to reach the peak value of bone parameters in the mandible was the same as the frontal bone but different from parietal bone. Of note, both the frontal bone and mandible are neural crest-derived, but parietal bone is mesoderm-derived, and this may contribute to the similarity and difference among these bones.

### Postnatal cranial base development

The cranial base is a supporting platform for brain development and contains multiple growth centers to drive both cranial and facial anterior-posterior growth (Thorogood, 1988). The ISS and SOS are two synchondroses located at the center of cranial base and are important centers of longitudinal growth in the skull, playing critical roles in upper face and cranial vault development. The cranial base of C57BL/6NCrl mice were evaluated by μCT to assess the dynamic morphological changes and fusion of the ISS and SOS as well as the bone parameter changes of pre-sphenoid, basi-sphenoid, and basi-occipital bones which are separated by the ISS and SOS.

**Figure 12:**
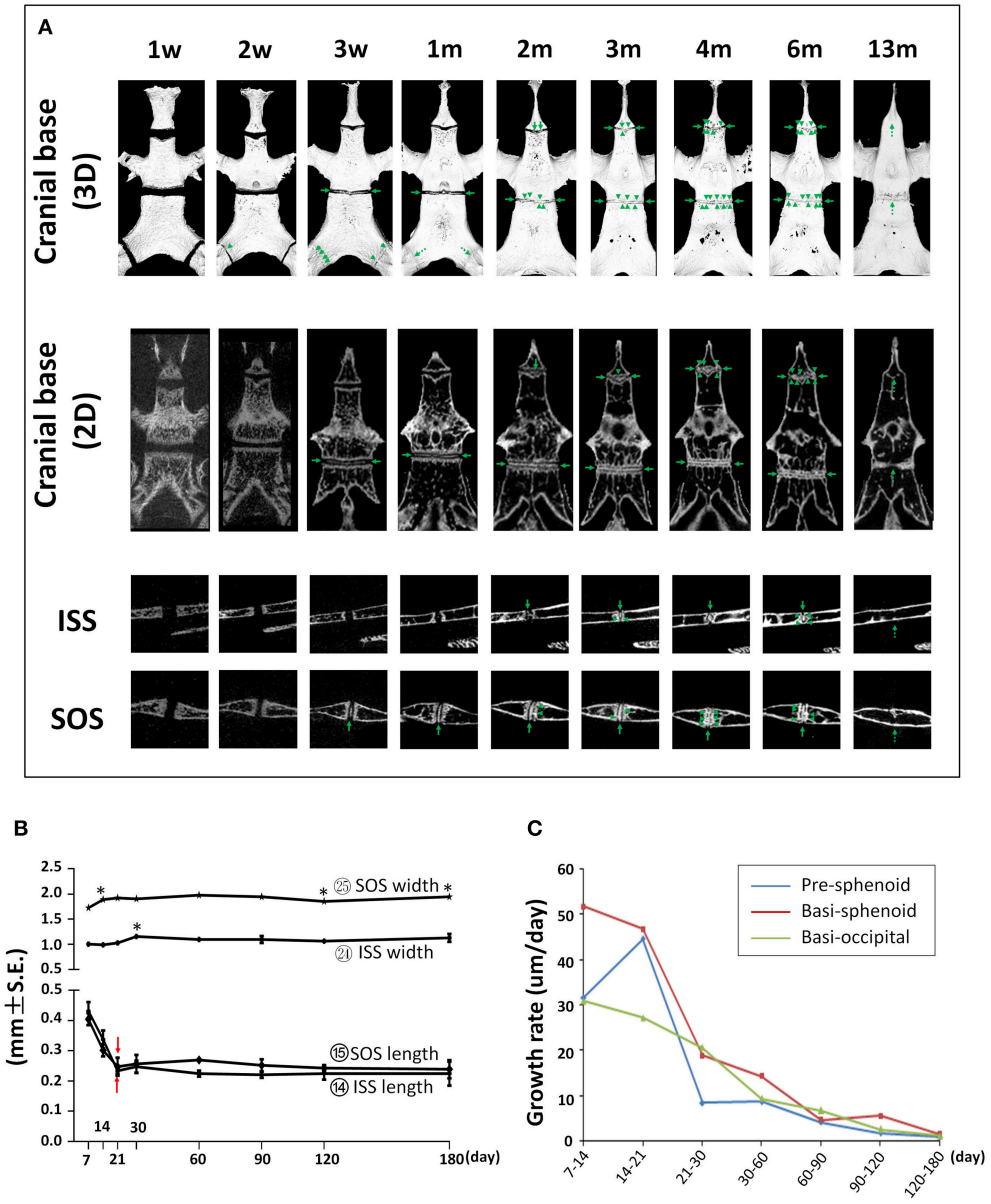
Postnatal changes of synchondroses in cranial base. **(A)** Representative μCT images of synchondroses in the cranial base in 3-D reconstruction and 2-D sections. Green arrows point to the radiopaque areas; green arrow heads point to the bony bridges; dashed green arrows point to the completely fused synchondroses. **(B)** Linear measurement (length and width) of inter-sphenoid synchondrosis (ISS) and sphenoid-occipital synchondrosis (SOS). ^*^Represents significant change between indicated data point and immediate earlier time point and red arrows indicate significant changes between all the neighboring time points before that time point (Tukey's multiple comparison test, *p* < 0.05). **(C)** Postnatal growth rate of pre-sphenoid, basi-sphenoid, and basi-occipital bone during the designated age intervals.

First, we determined the developmental dynamics of cranial base synchondroses. The ISS remained patent up to P60; however, small radiopaque areas can be seen within the central region of the ISS in 75% of the mice (Figure 12A and Figure S2). By P90, all animals had bony bridging between the caudal and rostral margins of the ISS. These incidences of bony bridging became more and more pronounced as mice aged, and the ISS was completely fused sometime between P180 and P390 (Figure 12A and Figure S2). Between P21 and P30, there was a 13% increase in the width of the ISS followed by a small but statistically significant 5% decrease at P60 compared to P30 (Figure 12B; circled 24). The A-P length of the ISS had a nearly 50% decrease from P7 to P21 and remained constant afterwards prior to its complete fusion (Figure 12B; circled 14). The SOS remained patent up to P30 and a small radiopaque area can be seen within the central region of the SOS in all mice at P21 (Figure 12A and Figure S2). By P60, all animals had bony bridging between the caudal and rostral margins of the SOS. These incidences of bridging became more and more pronounced over time and the SOS structure completely disappeared at P390 (Figure 12A and Figure S2). The SOS is 64–80% wider than the ISS. There was a 10% increase between P7 and P14, a 4% increase between P30 and P60, and a 5% decrease between P90 and P120 in SOS width (Figure 12B; circled 25). The A-P length of the SOS is comparable to that of the ISS. Similar to the ISS, there was a 40% decrease in the SOS length between P7 and P21 and the SOS length remained constant afterwards until its complete fusion (Figure 12B; circled 15). The exooccipital-basioccipital synchondrosis was patent at P7, but the bony bridging started to appear at P14 in most mice. The bony bridging became more evident at P21 and 100% of the exooccipital-basioccipital synchondrosis completely fused at P30 (Figure 12A and Figure S2).

**Figure 13:**
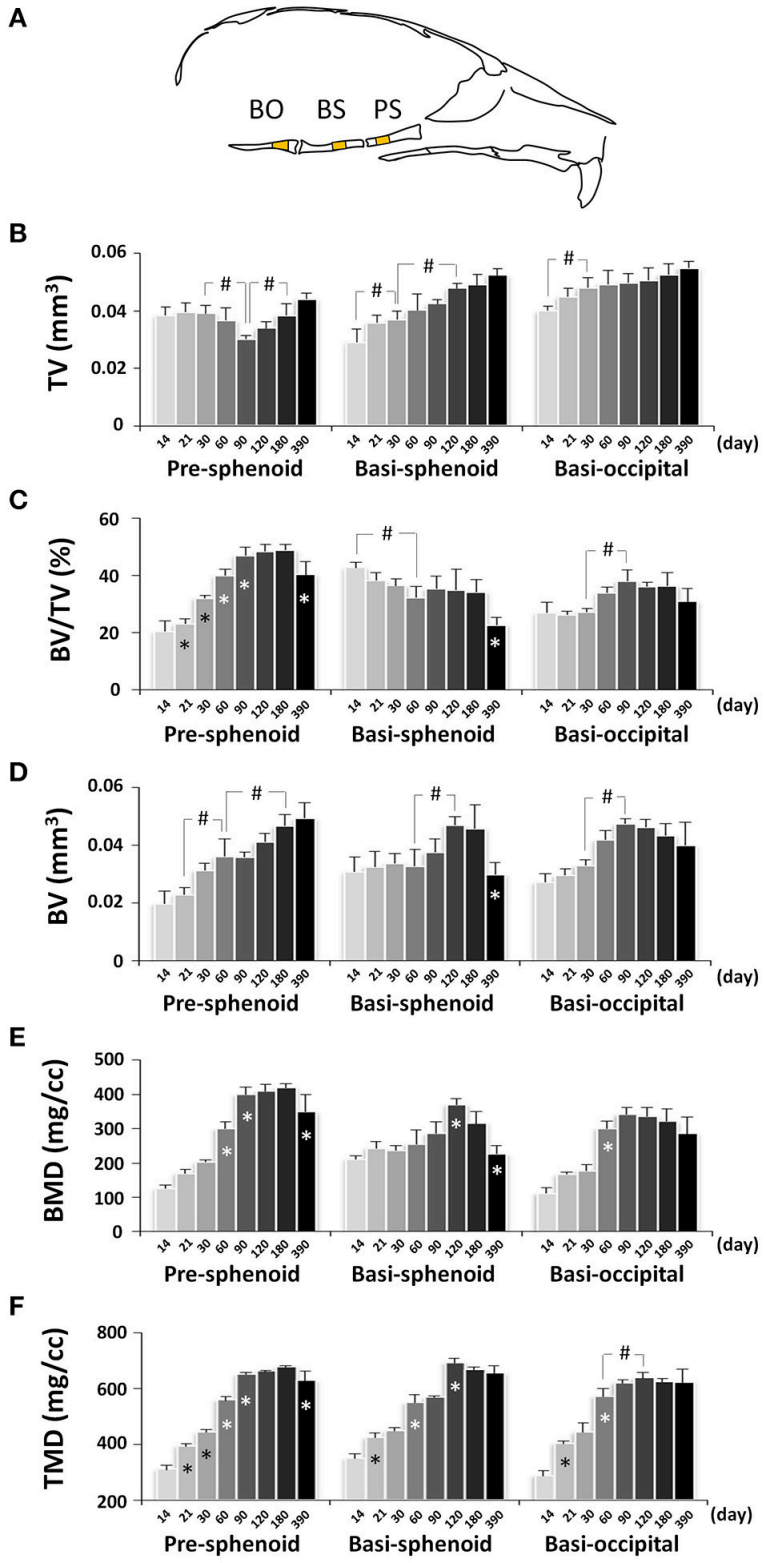
μCT analysis of bone parameters of cranial base bones. **(A)** Diagram of mouse skull at lateral view showing the measured area in pre-sphenoid (PS), basi-sphenoid (BS), and basi-occipital bone (BO). **(B–F)** μCT analysis of bone parameters (thickness, bone volume/tissue volume, bone volume, bone mineral density, and tissue mineral density, respectively) in cranial base bones with age. ^*^Represents significant change between indicated data point and immediate earlier time point; ^#^Represents significant change between indicated data points (Tukey's multiple comparison test, *p* < 0.05).

Next, we performed quantitative μCT analysis of bone parameters of pre-sphenoid, basi-sphenoid, and basi-occipital bones (Figure 13A and Figure S1B). Cranial base bones are formed through endochondral ossification, which is different from the calvaria, face and mandible that are formed through intramembranous ossification. The total volume (size) of the pre-sphenoid bone, which is derived from neural crest, was smallest at P90 and continuously increased afterwards (Figure 13B), reflecting a unique remodeling pattern. The BV/TV of the pre-sphenoid continued to increase until it plateaued from P90 to P180, with a drop at P390 (Figure 13C). As a result of the increase in BV/TV, the bone volume of the pre-sphenoid bone continued to increase (Figure 13D). The BMD of the pre-sphenoid bone followed a similar pattern to that of the BV/TV (Figure 13E). The TMD of the pre-sphenoid, a parameter reflecting the material density of bone itself, continued to increase until it peaked at P90, but it decreased significantly at P390 (Figure 13F). The basi-sphenoid is a bone of dual origin and the site chosen for measurement is of neural crest origin. The basi-sphenoid had a very different dynamic change in volume (size) compared to the pre-sphenoid, and it continued to increase (Figure 13B). The bone volume (Figure 13D), BMD (Figure 13E), and TMD (Figure 13F) of the basi-sphenoid all peaked at P120. However, the BV/TV of the basi-sphenoid bone had a gradual decrease from P14 to P60, and a significant decrease from P180 to P390 (Figure 13C). Noticeably, the BV, BV/TV, BMD, and TMD of the basi-sphenoid started the decline after P180. At P390, the basi-sphenoid had a 36% decrease in bone volume compared to a peak value at P120, which was contributed to by a 42% decrease in BV/TV and a 5% decrease in TMD. The decrease in bone volume of the basi-sphenoid after C57BL/6 female mice reached adulthood is most significant among all examined craniofacial bones. Mesoderm-derived basi-occipital bone had a steady increase in tissue volume during first month of age (Figure 13B), a continuous increase in BV/TV (Figure 13C), BV (Figure 13D), BMD (Figure 13E), and TMD (Figure 13F) until they peaked at P90 or P120.

## Discussion

Cranial skeletogenesis is a unique process from that in the rest of the body. The development of craniofacial bones and skull shape is a coordinated process. Our data showed that during the first month after birth, the head, body, tail, femur, and tibia had the fastest growth in length. Interestingly, the ratio between the length of the head and other parts of the body had the greatest decrease during this period of time, indicating that the head grows less in the anterior-posterior dimension than other parts of the body in length at early postnatal stages of female C57BL/6NCrl mice. The head of female C57BL/6NCrl mice is ~38% of the total body length at birth and ~28% at P90, and remained at a similar value afterwards. This trend is very similar to humans, in which the head is ~25% of the total body length at birth and ~15% in adults (Huelke, 1998). Conceptually, the relative ratio in length can be a simple index to identify and quantify craniofacial developmental abnormalities using other parts of the body such as the total body length as an internal reference.

Our data showed that the greatest growth in A-P, transverse, and vertical dimensions in craniofacial regions of female C57BL/6NCrl mice occurred during the first 3 weeks after birth. The growth continued after 3 weeks of age with much slower velocity. Despite the continuous increase in body weight, the length of both the femur and tibia of female C57BL/6NCrl mice had no further increase after P120, indicating that they stopped linear skeletal growth at P120. Similar to limb bones, most of the linear measurements in the craniofacial region had no change after P120, but there are indeed some changes at later developmental stages. In the A-P dimension, the facial length had a 4% increase in association with a 6% increase in nasal bone length between P180 and P390 (Figure 3); occipital bone, intraparietal bone, and frontal bone also had small A-P dimension change after P120; the cranial length/facial length ratio peaked at P390. In vertical dimension, the middle-cranial height (Figure 5; circled 28) had a 5% increase in an association with a 4% increase in cranial cavity area (Figure 9) between P120 and P390. Thus, the craniofacial morphology in female C57BL/6NCrl mice is mostly established at 4 months of age, but there are continuous changes in some dimensions at later developmental stages. This degree of late additional craniofacial growth is consistent with findings showing that the craniofacial skeleton of humans also continues to grow and change in adulthood (Pecora et al., 2008).

The development of the body skeleton has gender dimorphism. Differences in craniofacial skeletal development between male and female mice are expected, but it is unclear to what extent. While we were conducting this research, a recent report documented the postnatal craniofacial skeletal development in male C57BL/6J mice (Vora et al., 2016), which provided us a valuable reference to determine the differences between male and female mice with similar genetic background (Table S1). However, the genetic background of the mice in our study is slightly different from that report and the diet and housing conditions are also different. Yet, while we cannot know for sure if differences between the two strains of mice are due solely to gender, our data did show differential growth patterns between female C57BL/6NCrl and male C57BL/6J mice. In the A-P dimension, the contribution of the facial region to the overall skull length in female C57BL/6NCrl mice is significantly more than that in male C57BL/6J mice. Notably, the contribution of overlapped region between face and cranium to the A-P length of skull was significantly bigger in female C57BL/6NCrl mice. In addition, the maxilla length/mandible length ratio in female C57BL/6NCrl mice appears to be significantly bigger than that reported in male C57BL/6J mice, and this is consistent with the difference between female and male in humans, in which females have less mandibular growth (Ochoa and Nanda, 2004). In the transverse dimension, the width of the anterior facial region in female C57BL/6NCrl mice do not have the rapid increase phase as reported in male C57BL/6J mice, suggesting that female mice may have an earlier growth spurt in these regions. In particular, the frontal width at the middle of the cranial location had no change from P7 to P390 in female C57BL/6NCrl mice, but a rapid increase in the first month was reported in male C57BL/6J mice. In the vertical dimension, female C57BL/6NCrl mice had a decrease in cranial vault height between P21 and P30, and male C57BL/6J mice had a similar decrease but it was between P28 and P56. This distinction is another indication of the different developmental coordination seen between male and female mice. Altogether, our data indicate that female C57BL/6NCrl mice have generally a very similar craniofacial growth pattern compared to male C57BL/6J mice, but also have some unique features including a bigger contribution of the facial region to A-P dimension and a bigger maxilla/mandible length ratio. Greater facial convexity in females than males has also been reported for humans (Anic-Milosevic et al., 2008).

In contrast to the linear measurement of individual bones, the bone parameters of craniofacial bones had a continuous change over the time. The thickness of calvaria kept increasing between P120 and P390, but the BV/TV of these bones had opposite change. Compared to its peak tissue mineral density at P90, tissue mineral density of the frontal bone, a neural crest-derived bone, had a 6% decrease at P390. Similarly, tissue mineral density of parietal bone, a mesoderm-derived bone, had a 7% decrease at P390 compared to its peak value at P60. Thus, the bone quality of the calvaria may be compromised with aging despite an increase in bone volume. Similar to the frontal bone, the mandible is a neural crest-derived bone. Compared to the calvaria, the mandible has unique morphology and function and is subject to stress due to the mandibular movement and mastication. Interestingly, the thickness of the mandible decreased with age. However, the BV/TV was comparable at P390 compared to P60-P180, which is in contrast with a significant decrease in calvaria. Similarly, the tissue mineral density of the mandible at P390 was comparable to P120–P180. It is known that mechanical loading is an anabolic signal to bone (Robling and Turner, 2009; Barndt et al., 2015). The ability of the mandible to maintain BV/TV and tissue mineral density during later adulthood may be due to the beneficiary effect of mechanical loading due to its unique function in the oral cavity. Cranial base bones are formed through endochondral ossification (Nie, 2005). In contrast to calvarial bones, three cranial base bones in the midline had different dynamic changes in bone parameters, but all of them had stable BV/TV between P90 and P180 and a significant decrease in BV/TV at P390. The peak tissue mineral density in cranial base bones appeared later than in cranial vault bones and mandible. The significant differences in bone parameters among various craniofacial bones add to the remarkable topological and functional complexity of this region. Our data demonstrated the dynamic changes of craniofacial bone parameters during development; however, due to the limited sample size of this study, many changes in bone parameters were not yet statistically significant.

The cranial base has a major role in driving postnatal overall craniofacial growth (Parsons et al., 2015). Expansion of the cranial base occurs at the synchondroses, which are the counterpart of growth plate in long bones (Wei et al., 2016). The posterior cranial base is composed of the pre-sphenoid, basi-sphenoid, and basi-occipital bones which are separated by the ISS and SOS. At P7, the length of the posterior cranial base reached more than 60% of its length at P120, indicating an important role of early development. After the first week of age, there was still nearly 40% growth in the A-P dimension of the cranial base with the quickest growth between P7 and P21, followed by much slower growth afterwards and complete stop after P120. Unsurprisingly, the individual cranial base bones shared the similar growth trend as posterior cranial base (Figure 12C). The growth rate of the pre-sphenoid and basi-sphenoid bones between P7 and P14 in female C57BL/6NCrl mice studied here (~30–50 μm/day) is very similar to male C57BL/6J mice (~30–70 μm/day). Interestingly, in both male and female mice, the growth rate of the pre-sphenoid bone is higher between P14 and P21 than between P7 and P14. Similar to the decrease in growth rate of cranial base length, there was a fast decrease in the A-P length of the ISS and SOS during the first 3 weeks after birth, and they maintained the same length until the complete disappearance of the structure. Early postnatal cranial base growth is also an important component of human craniofacial development (Cendekiawan et al., 2010).

Similar to others, we found a radiopaque structure in the middle of the SOS at P21. This radiopaque region was termed “tethers” both in the growth plate of long bone (Chen et al., 2009; Lee et al., 2011) and the SOS (Lee et al., 2011). Histologically the tether in the SOS was shown to have an altered cellular morphology with mineralizing chondrocyte matrix (Vora et al., 2016). In female C57BL/6NCrl mice, we also observed this tether structure in the ISS at 2 months of age, which was not reported in the other study using male C57BL/6J mice (Vora et al., 2016). It is unknown whether this is due to gender or mouse strain differences. However, the tether structure in the synchondroses appears not to stop the growth of the cranial base since there was still considerable growth after its appearance. The elongation of the basi-occipital bone is contributed mainly by the SOS according to its location, and it still has more than 20% growth after the appearance of the tether structure. The exact role of these tether structures in synchondroses requires further studies.

Prior to the availability of the μCT-based craniofacial measurement, radiograph-based two-dimensional cephalometric linear and angular analysis of murine skulls has been developed long ago (Spence, 1940; Jolly and Moore, 1975; Engstrom et al., 1982). Radiograph-based cephalometry used in murine skulls is very similar to that used in human, which has practical and successful clinical applications. Although it was reported that certain landmarks are difficult to identify on two-dimensional radiograph of mouse skull (Bloom et al., 2006), radiograph-based cephalometry has been used successfully to identify morphometric changes in transgenic mice (Chung et al., 1997; McAlarney et al., 2001; Yagasaki et al., 2003; Simon et al., 2014; Badri et al., 2016). Thus, radiograph-based cephalometric analysis is an alternative to μCT-based craniofacial morphological measurement when the landmarks can be clearly identified. On the other hand, μCT-based craniofacial measurement has the advantage of high resolution and the ability to determine the morphology, quantity and quality of each individual bone.

In summary, we thoroughly documented the normative data of postnatal craniofacial skeletal development of female C57BL/6NCrl mice. The A-P length, width, and height of skull had the quickest growth in the first 3 weeks after birth and some parameters had continuous changes until postnatal day 390. The growth pattern of female C57BL/6NCrl mice is very similar to that of reported male C57BL/6J mice (Vora et al., 2016) but there are also differences (Table S1). Most of the bone parameters had the dynamic change throughout the whole development stages and many bone parameters such as calvarial thickness and BV/TV had the bigger increase after 3–4 weeks of age. These data can serve as reference standard for future quantitative work using this mouse strain.

## Ethics statement

All experimental procedures were carried out with the approval of the Institutional Animal Care and Use Committee at the University of Michigan (approval number: PRO00006354). All measures were taken to minimize the number of animals used.

## Author contributions

XW, NT, NH, MH, and FL conceived the study, XW and NT performed analyses, XW, NT, NH, MH, and FL contributed to data interpretation, XW and FL wrote the manuscript with NT, NH and MH contributing to critical revising.

### Conflict of interest statement

The authors declare that the research was conducted in the absence of any commercial or financial relationships that could be construed as a potential conflict of interest.
