# Supplementary figures and images for: Postnatal Craniofacial Skeletal Development of Female C57BL/6NCrl Mice
Source: Front Physiol. 2017 Sep 14;8:697. doi: 10.3389/fphys.2017.00697 (PMC5603710; doi:10.3389/fphys.2017.00697)

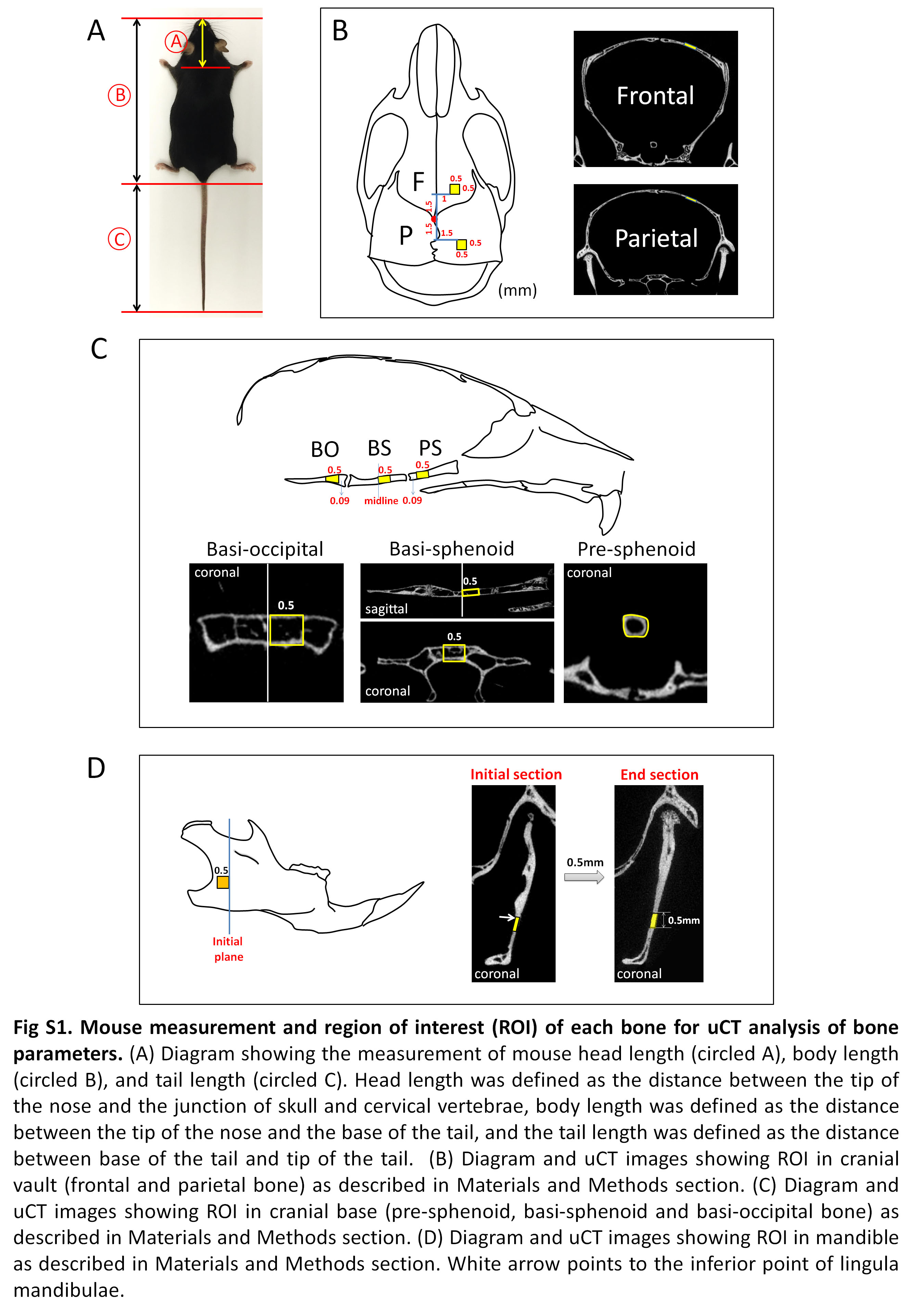

Supplement: Supplementary file 1 [file Image1.JPEG]

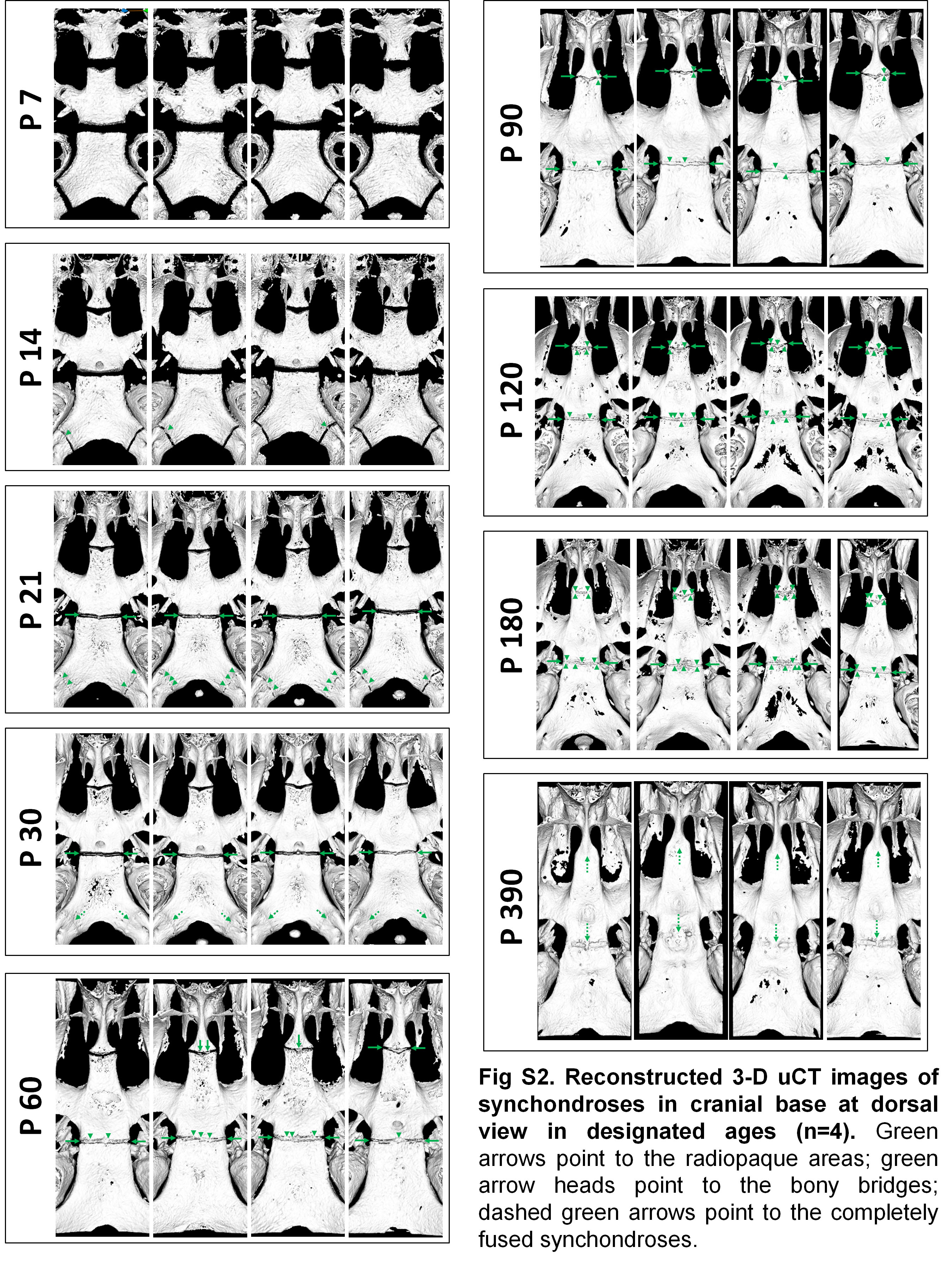

Supplement: Supplementary file 2 [file Image2.JPEG]
