# Supplementary material for: Postnatal Craniofacial Skeletal Development of Female C57BL/6NCrl Mice
Source: Front Physiol. 2017 Sep 14;8:697. doi: 10.3389/fphys.2017.00697 (PMC5603710; doi:10.3389/fphys.2017.00697)
Supplement: Supplementary file 3 [file Table1.DOCX]

**Supplementary Table 1. Gender dimorphism of craniofacial development between C57BL/6NCrl female mice and C57BL/6J male mice.**

| Dimension | Parameter | C57BL/6NCrl ♀ | C57BL/6J ♂ |
| --- | --- | --- | --- |
| A-P | Face contribution to skull | more | less |
|  | Overlap of face & cranium | bigger | smaller |
|  | Maxilla/Mandible ratio | higher | lower |
|  |  |  |  |
| Transverse | Anterior facial width | smaller increase before P30 | bigger increase before P28 |
|  | Frontal width | no significant change | significant increase (P7-P14) |
|  |  |  |  |
| Vertical | Cranial vault height | decrease from P21 to P30 | decrease from P28 to P56 |
